# Efficient CRISPR‐based genome editing using tandem guide RNAs and editable surrogate reporters

**DOI:** 10.1002/2211-5463.12437

**Published:** 2018-06-13

**Authors:** Wuqing Liu, Shifeng Li, Yunbin Zhang, Jinsong Li, Yiping Li

**Affiliations:** ^1^ State Key Laboratory of Cell Biology Shanghai Key Laboratory of Molecular Andrology CAS Center for Excellence in Molecular Cell Science Shanghai Institute of Biochemistry and Cell Biology Chinese Academy of Science University of Chinese Academy of Science Shanghai China

**Keywords:** all‐in‐one, CRISPR/Cas9, gene‐editing efficiency, surrogate reporter, tandem sgRNAs

## Abstract

Cleavage efficiency plays a key role in clustered regularly interspaced short palindromic repeat (CRISPR)‐based gene editing, particularly when the given guide RNA exhibits low cleavage activity. Here, we describe the packaging of tandem guide RNAs and single‐strand annealing‐based surrogate reporter cassettes into the CRISPR/CRISPR‐associated protein 9 vector, which increased gene‐editing efficiency by 4.94–6.31‐fold and simultaneously enriched the proportion of genetically modified cells. This strategy may substantially improve genome‐editing efficiency for demanding applications.

**Abbreviations**
Cas9: CRISPR‐associated protein 9
CRISPR: clustered regularly interspaced short palindromic repeat
*DAZL*: deleted in azoospermia‐like
DSBs: double‐strand breaks
FACS: fluorescence‐activated cell sorting
sg: single guide
SSA: single‐strand annealing

The clustered regularly interspaced short palindromic repeat (CRISPR)/CRISPR‐associated protein 9 (Cas9) system evolved in bacteria and archaea as a self‐defense mechanism against invading phage DNA 1, 2, 3. Molecular biologists have retooled the Cas9 nuclease into ‘molecule‐sized programmable scissors’ that, directed by a single guide (sg) RNA sequence, can precisely cleave the target at potentially any position in the genomes of diverse species 4, 5, 6, 7. This technology offers the power to manipulate genomes and holds great promise for clinical applications, such as for disease modeling and therapy 8, 9, as well as for altering the genomes of embryos or gametes 10, 11, 12, 13, 14.

Clustered regularly interspaced short palindromic repeat‐based genome editing remains in its infancy and requires further optimization. An ideal gene‐editing system would allow precise manipulation at any genomic locus with high efficiency and specificity, while facilitating subsequent identification and isolation of the genetically modified cells. While various prediction algorithms can help design sgRNAs that maximize on‐target efficiency and minimize off‐target events 15, 16, 17, 18, 19, many factors can influence whether the sgRNAs function as predicted 19, 20; these factors include expression levels of Cas9 and sgRNA 21, delivery efficiency 22, and characteristics of target cells 23. Furthermore, identifying edited cells is typically performed using fluorescence‐activated cell sorting (FACS) or antibiotic selection, which can lack sensitivity to detect the small proportion of Cas9‐positive cells that can be edited. Limited Cas9 cleavage efficiency and relatively insensitive selection approaches to isolate edited cells hinder the wider application of CRISPR‐based genome editing in biological research and clinical applications.

Among various approaches to enhancing cleavage efficiency 24, 25, 26, one of the more promising is to ensure adequate levels of sgRNA. A given sgRNA can show low or undetectable activity, and its recognition sequence requires a specific protospacer adjacent motif (PAM) at the end to initiate sgRNA‐mediated DNA recognition 27, 28. It means the choice of gRNA is often quite limited, especially in introducing a specific change at a specific site. Increasing the level of sgRNA can significantly improve the efficiency of on‐target cleavage 21.

One approach to achieving the sensitive selection of edited cells is based on the single‐strand annealing (SSA) DNA repair pathway. This pathway involves annealing of repeat sequences that flank a double‐strand break (DSB). The process is initiated when a DSB occurs between two repeated sequences oriented in the same direction; the subsequent bridging of the DSB leads to deletion of one of the repeats 29, 30. Using an SSA‐based surrogate reporter provides a robust, unbiased indicator of CRISPR editing performance and enriches for edited cells 31, 32.

Here, we combine both of these approaches in an ‘all‐in‐one’ strategy in which an SSA‐based CRISPR/Cas9 vector, Cas9 nuclease, sgRNA, and surrogate reporter are copackaged to provide a simplified workflow offering more efficient cleavage and enrichment of edited cells. This strategy was validated by targeting the deleted in azoospermia‐like (*DAZL*) gene, and the results suggest that ‘all‐in‐one’ editing can greatly simplify and expedite the CRISPR workflow, as well as maximize gene‐editing efficiency even at sites that are difficult to edit.

## Results and Discussion

### SSA‐based ‘all‐in‐one’ vector system

**Figure 1:**
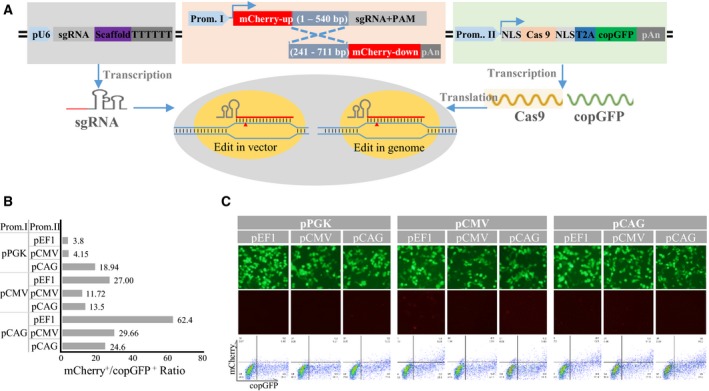
The SSA‐based ‘all‐in‐one’ CRISPR/Cas9 system. (A) The system comprises three expression cassettes: sgRNA, a SSA‐based mCherry protein, and a fusion of copGFP and Cas9 joined by a bridging peptide 2A. Each cassette is driven by a different promoter. (B) Quantitation of each promoter's activity using FACS. (C) Examination of copGFP and mCherry expression using fluorescence microscopy. Promoters can be replaced, such as when certain promoters are known to be more active in the target cells.

The vector backbone is derived from the pX330 vector, which has numerous, well‐positioned restriction enzyme sites and lacks many elements unnecessary for CRISPR‐based editing that inflate vector size. In addition, the vector contains three basic components required for genome editing: (a) a custom‐designed sgRNA cassette expressed off the U6 promoter, (b) two truncate encoding mCherry fragments designed to detect DSB‐induced SSA events at the target site, and (c) an expression cassette encoding a fusion of copGFP and Cas9 bridged by peptide 2A (Fig. 1A). The two mCherry fragments share a 0.3‐kb region of homology. The target sequence is inserted between the two split mCherry genes, and an in‐frame stop codon inserted between the mCherry‐up sequences prevents possible readthrough of the truncated mCherry gene. If a Cas9‐based DSB lies between two repeat sequences, it can lead to SSA‐mediated repair, ultimately leading to a deletion of one of the repeats. In this way, both mCherry and copGFP proteins will be produced when SSA occurs, whereas only copGFP will be expressed if SSA does not occur. The fluorescent signal of copGFP can be used to measure transfection efficiency and identify Cas9‐positive cells. The fluorescent signal of mCherry can be used to measure the efficiency of on‐target mutations and select edited cells using FACS.

The expression cassettes in the all‐in‐one vector include multiple promoters to allow users to select the most appropriate plasmids for robust expression and efficient cleavage. Experiments with empty vectors and four promoters (EF1α, CMV, CAG, and PGK) showed that the promoters exhibited quite different, dynamic properties. In the case of the Cas9‐copGFP expression cassette, the EF1α promoter drove robust expression of copGFP and gave results similar to those with the CMV and CAG promoters. In the case of the SSA‐based surrogate reporter expression cassette, signal from mCherry was observed in HEK 293T cells at 48 h after transfection with mock vector containing CAG, CMV, and PGK promoters (Fig. 1B,C). This may be due to low levels of leakage or recombination–deletion from the mock vector. The ratio of copGFP to mCherry was lowest with the PGK promoter and highest with the CAG and CMV promoters; this may reflect differences in promoter activity. To minimize background noise and increase signal‐to‐noise ratio, the PGK promoter was used to drive SSA‐based expression of mCherry, the EF1α promoter was used to drive expression of the Cas9‐peptide 2A‐copGFP fusion, and the U6 promoter was used to drive expression of sgRNA.

### Detection and on‐target efficiency of human editing events with the ‘all‐in‐one’ system

**Figure 2:**
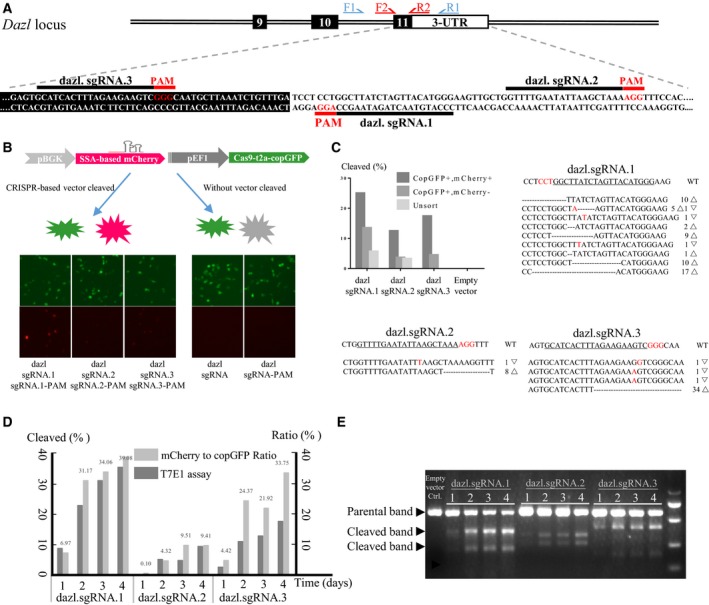
Schematic of the human *DAZL* gene and sequences around the target locus for generating functional knockouts using the SSA‐based ‘all‐in‐one’ system. (A) The diagram shows the DAZL locus and positions of sgRNAs. Exon coding sequences are shown in black boxes; UTRs, in white boxes. The sgRNA target is indicated in black; the PAM, in red. Arrows indicate the locations of nested PCR primers. F1 and R1 refer to the forward and reverse outer primers; F2 and R2, to the forward and reverse inner primers. (B, C) At 2 days after transfection, FACS was used to select copGFP/mCherry dual‐positive cells and copGFP‐positive/mCherry‐negative cells. The sorted cell populations were assessed for mutations using a mismatch detection assay, followed by Sanger sequencing of clonal amplicons to determine specific mutation events. Triangles indicate deleted bases; inverted triangles, insertions or mutations. (D, E) Time course showing variation in the mCherry: copGFP ratio and efficiency of DAZL editing.

We examined whether the percentage of copGFP‐positive cells that were also mCherry‐positive correlated with the efficiency of Cas9‐induced indels at endogenous loci. We set up editing reactions with three sgRNAs predicted to show different cleavage activities (dazl.sgRNA.1, dazl.sgRNA.2, dazl.sgRNA.3). These sgRNAs target the sequence between the last exon and the 3′ UTR of the endogenous human *DAZL* gene and thereby guide Cas9‐mediated cleavage (Fig. 2A). All‐in‐one vectors were constructed containing each sgRNA and its target site sequence. A negative‐control vector was constructed containing only the target site sequence. Cells were transfected and allowed to undergo genomic editing for 2 days, after which they were sorted based on fluorescence to isolate the copGFP‐positive/mCherry‐negative population and the copGFP/mCherry dual‐positive population. Genomic DNA was harvested and amplified by nested PCR; the amplicons were digested using T7 endonuclease I or Sanger‐sequenced.

At 48 h after transfection, transfection with dazl.sgRNA.1 led to a larger population of mCherry‐positive cells than transfection with the other sgRNAs, based on fluorescence microscopy and FACS. In the T7 endonuclease I assay, proportions of copGFP/mCherry‐positive cells were 24.79% with dazl.sgRNA.1, 12.26% with dazl.sgRNA.2, and 17.18% with dazl.sgRNA.3. The respective proportions of copGFP‐positive/mCherry‐negative cells were 13.25%, 3.44%, and 4.29% (Fig. 2B,C). The number of editing events increased with longer culture time (Fig. 2D,E). Thus, dazl.sgRNA.1 led to more genome editing than the other sgRNAs, consistent with mCherry expression.

These results indicate the mCherry expression is a reliable indicator of the efficiency of on‐target mutations achieved using our ‘all‐in‐one’ system. They also indicate that this system facilitates assessment of sgRNA and Cas9 performance, which may accelerate guide screening and facilitate identification of edited cells.

### Incorporation of multiple copies of sgRNA to increase cleavage efficiency

**Figure 3:**
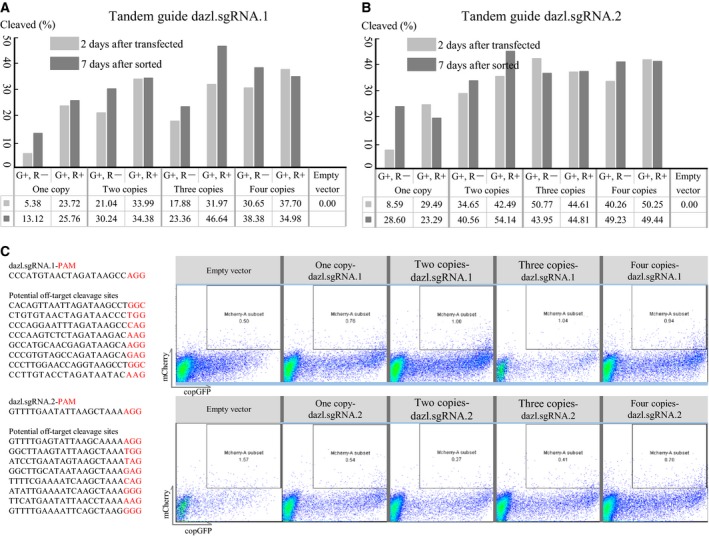
Effect of sgRNA dosage in the ‘all‐in‐one’ system and measurement of off‐target cleavage. (A, B) Comparison of editing efficiencies obtained with ‘all‐in‐one’ vectors encoding one or more copies of sgRNAs targeting the *DAZL* gene. (C) Measurement of cleavage of off‐target sites predicted to be potential cleavage sites based on the indicated sgRNAs targeting the *DAZL* gene.

In CRISPR‐based mutagenesis, Cas9 nuclease requires a PAM sequence adjacent to the sgRNA. The PAM sequence can be positioned at several locations for any given target site, so Web‐based prediction algorithms are usually used to identify locations more likely to be cleaved efficiently and specifically. In some cases, only one PAM site may be available, and it may be predicted to lead to inefficient or undetectable cleavage. This raises the question of how to achieve the desired editing efficiency independent of sequence‐based activity 33, 34. One possibility is to optimize sgRNA expression: Higher sgRNA levels can lead to more efficient cleavage. We reasoned that, as vectors can be designed to produce multiple sgRNAs for simultaneous editing at multiple target sites, perhaps we could simply encode repeats of the same sgRNA in our ‘all‐in‐one’ system to boost sgRNA expression and thereby cleavage efficiency. Therefore, we designed one novel all‐in‐one vector containing two, three, or four copies of an sgRNA expression cassette to boost sgRNA expression and transfer sequence‐based activity into quantitation‐based activity to improving sgRNA performance. At 2–3 days after transfection, all‐in‐one vector encoding two copies of the sgRNA led to a larger proportion of on‐target edited cells than a vector carrying only one copy, two copies with surrogate reporter cassettes can increase gene‐editing efficiency by 6.31‐fold with dazl.sgRNA.1 and 4.94‐fold with dazl.sgRNA.2. No difference was observed after prolonging the incubation period or encoding three or four copies of the sgRNA in the vector (Fig. 3A,B). Indicate that encoding two copies of the sgRNA can maximize the gene‐editing efficiency within the shortest time.

### Assessment of off‐target cleavage

A major challenge in using CRISPR/Cas9 for gene editing is the high incidence of genome cleavage at off‐target sites 5, 20, 35, 36. The possibility that sgRNAs may lead to off‐target cleavage at sites showing partial homology is always present, and may even be worse when expressing multiple copies of the sgRNA. Therefore, we measured the probability of off‐site cleavage with our ‘all‐in‐one’ system carrying multiple copies of dazl.sgRNA.1 and dazl.sgRNA.2. We synthesized a contiguous series of potential off‐target site sequences predicted for dazl.sgRNAs (Fig. 3C), which we inserted between SSA‐reporter cassettes. The resulting vector was transfected into HEK 293T cells, and mCherry‐positive populations were compared. The results suggest that the probability of off‐site cleavage is similar for vectors carrying one or two sgRNA copies.

### Further validation of the ‘all‐in‐one’ system with mouse genes

**Figure 4:**
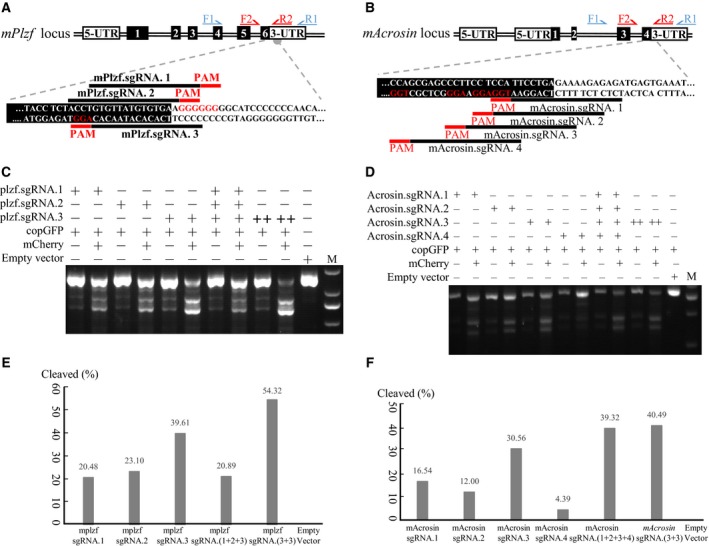
Application of the ‘all‐in‐one’ system to knock out genes involved in mouse spermatogenesis. The chromosomal locus and position of sgRNAs are shown for (A) *PLZF*, (B) *ACR* genes. Coding sequences are shown in black boxes; 3′ UTR, in white boxes. At 3 days after transfection, B16 cells were subjected to fluorescence‐activated sorting to identify copGFP/mCherry dual‐positive and copGFP‐positive/mCherry‐negative populations. These sorted populations were analyzed in a mismatch detection assay to determine specific mutation events in the genes: (C, E) *PLZF*, (D, F) *ACR*.

To assess the performance of the ‘all‐in‐one’ gene‐editing system against additional targets, we programmed the vector with sgRNAs against the following mouse genes involved in spermatogenesis: The *PLZF* gene encodes promyelocytic leukemia zinc finger (also ZBTB16), a selective marker that favors the renewal of spermatogonial stem cells over their differentiation; and the *ACR* gene encodes acrosin, the major protease in the acrosome of mature spermatozoa. Several sgRNAs were screened for their ability to induce cleavage at a target site between the last exon and the 3′ UTR of these genes (Fig. 4A,B).

A vector carrying a single copy of sgRNA.3 against the *PLZF* gene yielded an editing efficiency of 39.61% in mouse B16 melanoma cells at 3 days after transfection, compared to 20.48% for sgRNA.1 and 23.10% for sgRNA.2. Editing efficiency increased to 54.32% when two copies of sgRNA.3 were used. Conversely, a vector carrying one copy each of sgRNA.1, sgRNA.2, and sgRNA.3 resulted in editing efficiency of only 20.89% (Fig. 4C–E), consistent with the fact that one sgRNA can bind the sense strand while other sgRNAs bind the antisense strand, decreasing editing efficiency.

As in the *PLZF* case, using a vector carrying two copies of the sgRNA.3 against *ACR* led to editing efficiency of 40.49%, which was 1.32‐fold more efficient than a vector carrying only one copy (Fig. 4D,F). In contrast to the *PLZF* case, a vector carrying one copy each of sgRNA.1, sgRNA.2, sgRNA.3, and sgRNA.4 led to editing efficiency of 39.32%, which was similar to that obtained with two copies of sgRNA.1.

These results with *PLZF* and *ACR* suggest that using more sgRNAs improves the efficiency of gene editing with the ‘all‐in‐one’ system.

Our findings with vectors simultaneously encoding multiple different sgRNAs against the same gene (‘cocktail sgRNAs’) suggest that these sgRNAs should be carefully designed to avoid unwanted results. If sgRNAs in the cocktail target different strands (some sense, others antisense), Cas9 cleavage may be blocked. If all sgRNAs target the same strand (sense or antisense), Cas9‐mediated cleavage can occur efficiently, even when sequences overlap. This is because when one sgRNA molecule binds the target sequence, other sgRNA molecules can no longer bind to it, allowing Cas9 to complex with the sgRNA and genomic DNA.

## Conclusion

The power of CRISPR/Cas9 for genomic editing will undoubtedly make it a focus of continued optimization for basic and clinical applications. Here, we demonstrate that an ‘all‐in‐one’ CRISPR/Cas9 system that contains two tandem copies of the sgRNA or cocktail sgRNAs targeting the same strand in order to promote cleavage activity, as well as an SSA‐based ‘editable reporter’ to visually indicate editing efficiency, can provide a simplified platform for modifying genes and selecting individual cells in which editing has been successful. This system may help accelerate the development of CRISPR/Cas9 for diverse biomedical applications.

## Materials and methods

### Design and synthesis of sgRNAs

The sgRNAs were designed using the CRISPR tool (http://crispr.mit.edu), and their sequences as well as the target sequences are listed in Table S1.

### Construction of the ‘all‐in‐one’ vector

‘All‐in‐one’ vectors were constructed using a pX330 backbone. Digestion–ligation or seamless cloning techniques were used to subclone sgRNA/Cas9 cassettes and other components. Briefly, empty vectors were used as template to generate a PCR product, which was cloned into a suitably digested vector using the in‐fusion technique. The sgRNAs and target sequences were cloned into vectors using digestion–ligation. Cassettes carrying multiple copies of sgRNAs were constructed using in‐fusion cloning.

### Cell culture and transfection

HEK 293T and mouse B16 melanoma cell lines were maintained in Dulbecco's modified Eagle's Medium supplemented with 10% fetal bovine serum, 2 mm GlutaMAX (Life Technologies, Carlsbad, CA, USA), 100 U·mL^−1^ penicillin, and 100 μg·mL^−1^ streptomycin (Life Technologies). Cultures were incubated at 37 °C with 5% CO_2_. Cells were seeded into 24‐well plates 1 day prior to transfection and then transfected using Lipofectamine 3000 (Life Technologies) following the manufacturer's recommended protocol.

### Fluorescence‐activated cell sorting

At 2 or 3 days post‐transfection, cells were resuspended, sorted based on copGFP or mCherry expression into 96‐well plates using the BD Influx cell sorter (BD Biosciences, San Jose, CA, USA), and expanded. Depending on the experiment, certain cell populations were maintained and expanded to accumulate editing events.

### Direct nested PCR of sorted cells and detection of on‐target mutations

To examine on‐target editing events, target sites within sorted cells were amplified using direct nested PCR and KOX FX neo polymerase (Toyobo, Tokyo, Japan). In this reaction, the genomic region encompassing the sgRNA target sequence was amplified using ‘external/internal’ primer pairs (Table S2). The PCR product was analyzed in a commercial T7 endonuclease I assay (Vazyme, Nanjing, China) following the manufacturer's recommended protocol, or it was Sanger‐sequenced. For the endonuclease assay, PCR product (approximately 200 ng) was mixed with 1 μL 10× buffer and DNA‐free water to a final volume of 10 μL, and then subjected to the following re‐annealing protocol to enable heteroduplex formation: 95 °C for 5 min, ramp from 95 °C to 85 °C at −2 °C·s^−1^, ramp from 85 °C to 25 °C at −0.1 °C·s^−1^, and holding at 4 °C for 1 min. Re‐annealed products were digested with T7 endonuclease 1 at 37 °C for 15 min, analyzed by agarose gel electrophoresis, and quantified based on relative band intensities.

## Author contributions

WL and YL conceived and designed the study; WL, SL, and YZ performed experiments; WL, JL, and YL analyzed data; WL wrote the manuscript; all authors participated in the revision of the initial manuscript and approved the final manuscript.

## Supporting information


**Table S1.** List of sgRNAs and target site primers used in the study.

**Table S2.** Primers used in the T7 endonuclease I assay.
