# Supplementary material for: Efficient CRISPR‐based genome editing using tandem guide RNAs and editable surrogate reporters
Source: FEBS Open Bio. 2018 Jun 13;8(7):1167–75. doi: 10.1002/2211-5463.12437 (PMC6026697; doi:10.1002/2211-5463.12437)
Supplement: Supplementary file 1 — Table S1. List of sgRNAs and target site primers used in the study. [file FEB4-8-1167-s001.pdf]

Supplementary Table 1 List of sgRNAs and target site primers used in the study

Target gene	Guide	Sequence
Human *DAZL*	dazl.sgRNA.1-F	CACCGCCCATGTAACTAGATAAGCC
	dazl.sgRNA.1-R	AAACGGCTTATCTAGTTACATGGGC
	dazl.sgRNA.2-F	CACCGTTTTGAATATTAAGCTAAA
	dazl.sgRNA.2-R	AAACTTTAGCTTAATATTCAAAAC
	dazl.sgRNA.3-F	CACCGCATCACTTTAGAAGAAGTC
	dazl.sgRNA.3-R	AAACGACTTCTTCTAAAGTGATGC
	dazl.sgRNA.1-AGG-F	CACCGCCCATGTAACTAGATAAGCCAGG
	dazl.sgRNA.1-AGG-R	AAACCCTGGCTTATCTAGTTACATGGGC
	dazl.sgRNA.2-AGG-F	CACCGTTTTGAATATTAAGCTAAAAGG
	dazl.sgRNA.2-AGG-R	AAACCCTTTTAGCTTAATATTCAAAAC
	dazl.sgRNA.3-GGG-F	CACCGCATCACTTTAGAAGAAGTCGGG
	dazl.sgRNA.3-GGG-R	AAACCCCGACTTCTTCTAAAGTGATGC
Mouse *PLZF*	plzf.sgRNA.1-F	CACCGCCTGTGTTATGTGTGAAGGG
	plzf.sgRNA.1-R	AAACCCCTTCACACATAACACAGGC
	plzf.sgRNA.2-F	CACCGCCCAACACATGGTAGAGCAG
	plzf.sgRNA.2-R	AAACCTGCTCTACCATGTGTTGGGC
	plzf.sgRNA.3-F	CACCGCCCCCCTTCACACATAACAC
	plzf.sgRNA.3-R	AAACGTGTTATGTGTGAAGGGGGGC
	plzf.sgRNA-NGG-F	AACGGATCCTCCATGCAGAAACACATGA
	plzf.sgRNA-NGG-R	ATCAAGCTTTCCACTTTGTCTGGTTCTAG
Mouse *ACR*	acrosin.sgRNA.1-F	CACCGCATCTCTCTTTTCTCAGGAA
	acrosin.sgRNA.1-R	AAACTTCCTGAGAAAAGAGAGATGC
	acrosin.sgRNA.2-F	CACCGCTTTTCTCAGGAATGGAGGA
	acrosin.sgRNA.2-R	AAACTCCTCCATTCCTGAGAAAAGC
	acrosin.sgRNA.3-F	CACCGCTCTCTTTTCTCAGGAATGG
	acrosin.sgRNA.3-R	AAACCCATTCCTGAGAAAAGAGAGC
	acrosin.sgRNA-NGG-F	AACGGATCCAGTACAGTGGACCAAGGAACTAC
	acrosin.sgRNA-NGG-R	ATCAAGCTTGATGGAGGAAGATTTGGGTTGAG
