# Supplementary material for: Efficient CRISPR‐based genome editing using tandem guide RNAs and editable surrogate reporters
Source: FEBS Open Bio. 2018 Jun 13;8(7):1167–75. doi: 10.1002/2211-5463.12437 (PMC6026697; doi:10.1002/2211-5463.12437)
Supplement: Supplementary file 2 — Table S2. Primers used in the T7 endonuclease I assay. [file FEB4-8-1167-s002.docx]

Supplementary Table 2 Primers used in the T7 endonuclease I assay

| Genomic target | Primer | Sequence |
| --- | --- | --- |
| Human *DAZL* | dazl-F1 | AAGCCGTGGTTCCTATTAGATG |
|  | dazl-R1 | CTTGAACTGCTCTGCTCATACA |
|  | dazl-F2 | TGTTATCCCAGTGTTCAAGAATGCT |
|  | dazl-R2 | TGTTCACAGGTACTTGTTGGAGAAG |
| Mouse *PLZF* | plzf-F1 | GCATCGGCGGTGACTATTG |
|  | plzf-R1 | CAGGAAGTCGGTGCTAATCTC |
|  | plzf-F2 | CTACCTCTCCTCCTTACTGACTG |
|  | plzf-R2 | TTGGTCGACTCTTGCTCATGAGGGAG |
| Mouse *ACR* | acrosin-F1 | GCTGGCCTTTTGCTCGAATTCATTGCCCAAACCTGGTCC |
|  | acrosin-R1 | GTTAATCAGTCGTTGGTCGACATTCCTATTGGCCCCGCA |
|  | acrosin-F2 | GGACTACCTGGACTGGATTGC |
|  | acrosin-R2 | CATCAAGAGAACCGCCACTTAG |
